# The effect of a multimodal dementia prevention program involving community‐dwelling elderly

**DOI:** 10.1111/psyg.12790

**Published:** 2021-12-05

**Authors:** Hiroyuki Kajita, Kiyoshi Maeda, Tohmi Osaki, Yasumasa Kakei, Kavita U. Kothari, Yoji Nagai

**Affiliations:** ^1^ Faculty of Rehabilitation Kobe Gakuin University Kobe Japan; ^2^ Clinical & Translational Research Centre Kobe University Hospital Kobe Japan; ^3^ Division of Translational Science Kobe University Graduate School of Medicine Kobe Japan; ^4^ Department of Clinical Research Facilitation Institute for Advancement of Clinical and Translational Science, Kyoto University Hospital Kyoto Japan

**Keywords:** cognitive decline, dementia, preventive program, primary prevention

## Abstract

**Background:**

With the rapid increase in the average age of society, the number of people with dementia has increased in Japan. Thus, the need to prevent dementia is greater, and prevention programs have been implemented throughout Japan. This study aims to evaluate both the short‐term and the long‐term effects of a dementia prevention program on physical and cognitive function in community‐dwelling elderly.

**Methods:**

Cognitive and physical assessments were carried out at baseline for a sample including 57 elderly participants. The participants underwent an intensive training program lasting for 2 h per week for 10 days. After the last period of training, the assessment performed was reapplied. The outcome measures used to establish effectiveness were a Mini‐Mental State Examination, Five Cognitive Tests, a Cognitive Function Instrument, a Timed Up & Go Test, a grip strength evaluation, a Geriatric Depression Scale, an EQ‐5D and a Physical Activity Scale for the Elderly. Participants were then divided randomly into two groups: a booster group and a non‐booster group. The booster group received booster training every 3 months after the intensive training period, whereas the non‐booster group did not. Both groups were monitored every 6 months for approximately two and a half years after baseline assessment.

**Results:**

The Mini‐Mental State Examination, the subtests of the Five Cognitive Tests (attention, memory, language and reasoning) and the Timed Up & Go Test revealed a significant improvement after intensive training. For most of the outcome measures, the booster training showed no additional significant improvements.

**Conclusions:**

In this study, intensive training had a short‐term positive effect. Although the effect of the booster training was not clear, the functions of the elderly participants were found to be maintained during a follow‐up assessment. The study findings recommend conducting intensive training for the community‐dwelling elderly without follow‐up training.

## INTRODUCTION

Japan is experiencing both an ageing population and a decline in birth rate that is unparalleled in the rest of the world. It is estimated that the elderly (< 65) made up 27.7% of the population in 2017.^1^, ^2^ Japan can be said to be faced with a ‘super‐ageing’ society. Along with the rapid ageing of society, the number of people with dementia has increased.^3^ A similar trend is being seen in other countries, particularly in developed countries. In 2015, an estimated 47 million people were living with dementia worldwide, and this number is projected to triple by 2050.^4^

Alzheimer's disease (AD), which is the most common cause of dementia, is an incurable and progressive neurodegenerative disorder. At present, AD cannot be treated completely, although several medications such as acetylcholinesterase inhibitors can slow the disease's progression. In addition to pharmacotherapy, nonpharmacologic therapy is a potential pathway for treating dementia.^5^ People with dementia may benefit from nonpharmacologic approaches, including cognitively engaging activities, physical exercise and socialisation.^6^ However, as with pharmacotherapy, nonpharmacologic therapy cannot cure dementia. Consequently, there is an increasing need to prevent dementia, and many municipalities in Japan have launched a dementia prevention program (DPP).^7^ Effective dementia prevention strategies would provide substantial benefits by improving quality of life, prolonging independent life expectancy and reducing economic costs and social burdens.^8^

Multiple potential risk factors, such as depression, cardiovascular and cerebrovascular disease, diabetes, cognitive function, physical function, self‐rated health and lifestyle characteristics, are reported to be associated with the incidence of dementia.^8^, ^9^ Thus, the incidence of dementia can be reduced by exercising moderately in daily life to prevent lifestyle diseases. An important purpose of the DPP is to help the elderly understand the factors associated with the incidence of dementia and to live a daily life that carries less risk of developing dementia.

Although efforts to prevent dementia have grown popular over time, there are no proven modalities for preventing dementia.^10^ In 2016, Kobe City held a dementia prevention class for citizens in each city ward. Each class consisted of six programs. Other municipalities adopt a similar method, holding programs once or twice a week for approximately 10 days, after which the programs cease. Because dementia, particularly AD, is a neurodegenerative disease that progresses over the long‐term, adopting a long‐term approach to prevent and treat the disease is desirable. However, to continuously manage a DPP for a large number of citizens, a municipal office requires a large budget and significant human resources. Ideally, a DPP should be run more efficiently with limited resources.

In this study, we conducted intensive interventions for all participants and divided them into a booster training group (BTG) and a non‐booster training group (NonBTG). Then, we compared the cognitive and physical function changes between the two groups. Many previous intervention studies on dementia prevention compared the cognitive function changes between an intervention group and a non‐intervention group, but they did not compare the changes after conducting a common intensive DPP in all participants. The main aim of this study is to clarify the following two points: (i) short‐term effects of intensive training (once a week for 10 sessions); and (ii) longitudinal effects of regular booster training (once every 3 months) following intensive training and conducted over a 2‐year period.

## METHODS

### Participants

The sample population comprised community‐dwelling elderly aged at least 70 years who were flagged as being at high risk for mild cognitive impairment (MCI)/dementia because of unfavourable answers on the cognitive domain of the ‘Kihon Checklist’ in 2015 and consequently participated in a dementia prevention education program organised by Kobe City in 2016. The Kihon Checklist, which has been developed by the Japanese Ministry of Health, Labour and Welfare, is a simple 25‐item questionnaire used to identify frail citizens, and includes three items (Q18–20) on subjective cognitive function (the cognitive domain).^11^, ^12^ We deemed participants in the current study to be at risk for future dementia. We excluded from the study subjects who had been diagnosed with dementia by a medical doctor, in addition to subjects with obvious dementia symptoms (Mini‐Mental State Examination (MMSE) score of ≦ 23 at baseline evaluation), mental disorders and severe motor disabilities. We also excluded subjects who were absent from more than three intensive training sessions out of 10 and subjects who did not receive booster training every 3 months in the BTG.

### Study setting

The baseline assessment of cognitive and physical function for 57 elderly participants included the following elements: an MMSE, Five Cognitive Tests (Five Cog), a Cognitive Function Instrument (CFI), a Timed Up & Go Test (TUG), a grip strength evaluation, a Geriatric Depression Scale (GDS), an EQ‐5D, and a Physical Activity Scale for the Elderly (PASE). The intensive training program was conducted for 2 h per week for 10 days. After the last intensive training, the assessment performed at baseline was reapplied. Participants were then divided randomly into two groups: a BTG and a NonBTG. The BTG received booster training once every 3 months after the intensive training period, whereas the NonBTG did not. We monitored both groups every 6 months during follow‐up assessment. We conducted four follow‐ups (FU‐1–FU‐4) over approximately two and a half years.

DPP training sessions were held in a group for 2 h at a time. We conducted the DPP once a week for 10 sessions during the intensive training period, and once every 3 months as booster training for the BTG following the intensive training. The DPP comprised physical exercise (aerobic exercise, stretching, and strength training), cognitive training, dual task training, nutrition education by a dietitian and lectures on dementia. Aerobic exercise was performed for approximately 15 min in a standing or sitting position with music, and strength training was repeated with either a light or moderate load depending on the participant's physical function in consideration of their health risks. Cognitive training included the tasks such as working memory, calculation (the four basic arithmetic operations), spotting the difference between two similar pictures and reading aloud. As dual task training, exercise and cognitive tasks were performed simultaneously, such as calculation while walking. The lectures explained the factors related to the incidence of dementia and recommended physical and cognitively engaging activities, communication with neighbours, a well‐balanced diet and a moderate amount of sleep in daily life. The contents of the DPP were chosen from seemingly effective interventions for reducing cognitive decline in previous studies.^13^

We performed the baseline assessment in November 2017.

### Statistical analysis

The Mann–Whitney *U*‐test was used to compare the results of the assessments of the BTG and NonBTG participants at post‐intensive training (PIT). To investigate the effects of the intensive training, the respective outcome measures evaluated at baseline and PIT were compared using the Wilcoxon signed rank test.

We used a linear mixed‐effects model to analyse the effects of the booster training. Dependent variables were the scores of outcome measures for each assessment period (‘time’). Independent variables were ‘group’ (BTG or NonBTG) (a fixed factor) and a within‐subjects factor (a variable factor). We transformed the scores of outcome measures using the Box Cox transformation. For the outcome measures for which we observed a significant interaction using a liner mixed‐effects model, we conducted a pairwise comparison in each case using Bonferroni's correction. The significance threshold was set at *P* < 0.05. Statistical analysis was carried out by JMP®15 (SAS Institute Inc., Cary, NC, USA).

### Outcome measurements

The primary outcome measure for establishing effectiveness was the change of cognitive function using the MMSE score. Secondary outcome measures included the changes in Five Cog, CFI, TUG, grip strength, GDS, EQ‐5D and PASE. Details of the outcome measures are as follows.

The MMSE^14^ can be used to assess mental status both systematically and thoroughly. It is an 11‐question measure that tests five areas of cognitive function; these are orientation, registration, attention and calculation, recall and language. The maximum score is 30. A score of 23 or below is indicative of cognitive impairment.
The Five Cog^15^ was developed especially for older Japanese adults as a screening instrument to detect cognitive decline. It is a group assessment tool for cognitive functions that consists of five subtests (attention, memory, visuospatial, language and reasoning). A high score indicates better functioning.
The CFI^16^ is a screening tool for detecting early changes in the activities of daily living arising from cognitive decline. It comprises 14 questions that are related to reduced functional ability arising from cognitive impairment. The CFI score is calculated by ascribing 1 point for ‘Yes’, 0 points for ‘No’ and 0.5 points for ‘Maybe’. The CFI questionnaire includes ‘self’ and ‘partner’ versions; this study uses the ‘self’ version.
The TUG^17^ is a simple test used to assess a person's mobility and it requires both static and dynamic balance. It measures the time that a subject takes to rise from a chair, walk three metres, turn around, walk back to the chair and sit down.
The GDS^18^ is a screening test that is used to identify depression symptoms in older adults. The scale is a self‐reported instrument that adopts a ‘Yes/No’ format on a short form containing 15 questions.
EQ‐5D^19^ is a standardised measure of health status developed by the EuroQol Group to provide a simple, generic measure of health for both clinical and economic appraisal. It can be applied over a wide range of health conditions and treatments. The EuroQol Group provides two versions of the EQ‐5D with either three or five dimensions; for this study, we used the latter (EQ‐5D‐5L), which comprises five categories with five questions in each category. Depending on the pattern of answers, an individual's health status is given either as a single index value, ranging from zero to one, or death to perfect health. This study used only a single index value for statistical analysis.
7)The PASE,^20^ which is easy to administer and score, measures the level of physical activity in people aged 65 years and above. The instrument consists of self‐reported occupational, household and leisure activity items over a period of 1 week. The present study used the total PASE score.
We measured grip strength of the dominant hand of the participant in kilograms by using a Smedley‐type handheld dynamometer (GRIP‐D; Takei Scientific Instruments Co., Ltd., Japan).

### Ethical consideration

Our study protocol was approved by the ethics review committee of Kobe Gakuin University and the WHO ethics committee (ERC.0002899). The study was carried out in accordance with the provisions of the Declaration of Helsinki and Japan's ‘Ethical Guidelines for Medical and Health Research Involving Human Subjects.’

## RESULTS

**Figure 1:**
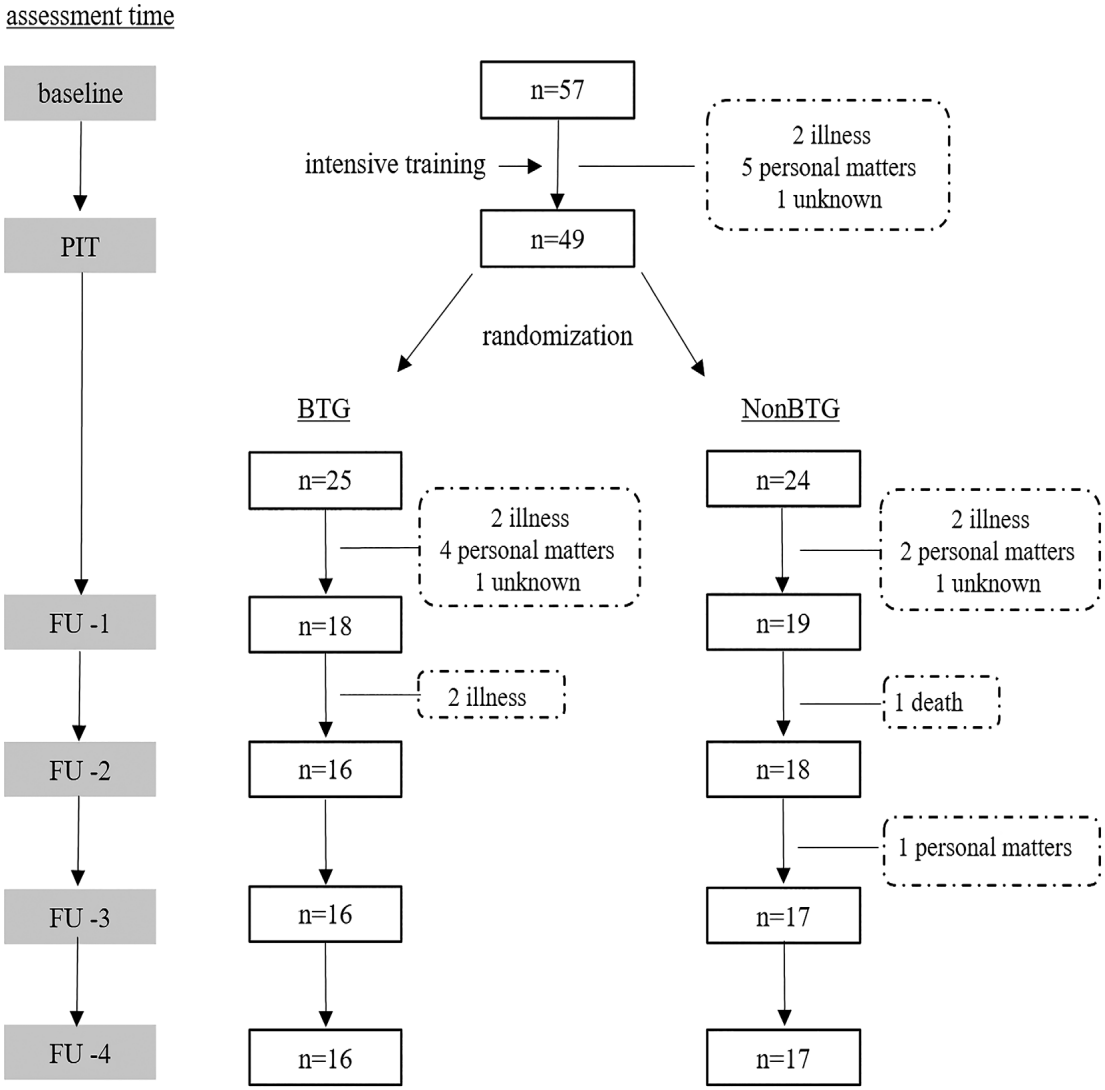
Study overview, showing the change in the number of participants in this study. BTG, booster training group; NonBTG, non‐booster training group; PIT, post‐intensive training; FU‐1, follow up 1; FU‐2, follow up 2; FU‐3, follow up 3; FU‐4, follow up 4.

This study involved 57 participants (21 male, 36 female) who underwent 10 sessions of intensive training. The mean (SD) age of the participants was 76.4 (2.7) years, and the mean years of education were 12.0 (2.3) years. We excluded from the project subjects who missed the intensive training three or more times. Forty‐nine participants received PIT assessment. We randomly assigned 25 participants to the BTG and 24 to the NonBTG. Sixteen participants dropped out during the follow‐up period, and the eventual number of participants at the FU‐4 assessment was 16 (6 male, 10 female) in the BTG and 17 (6 male, 11 female) in the NonBTG. The chief causes of the dropouts were personal matters and hospitalisation due to poor physical conditions (Fig. 1). The mean ages of the BTG and the NonBTG participants were 75.8 (3.2) years and 76.6 (2.4) years, respectively. There were no significant differences between the two groups in age (*P* = 0.39) and measurement results (Table 1).

**Table 1:** Assessment result of the post‐intensive training in the BTG and the NonBTG

	BTG (*n* = 16)	NonBTG (*n* = 17)	*Z* score	*P*‐value
MMSE	28 (26.3–29.0)	28 (27.0–30.0)	−1.03	0.30
Five Cog				
Attention	29 (24.3–33.0)	29 (23.5–30.0)	−0.33	0.75
Memory	14.5 (11.0–20.5)	17 0.0 (11.5–22.5)	−0.51	0.61
Visuospatial	7.0 (7.0–7.0)	7.0 (7.0–7.0)	0.00	1.0
Language	17.5 (15.0–21.8)	16.0 (13.5–20.0)	−1.24	0.26
Reasoning	12.0 (9.3–14.0)	11.0 (9.5–13.0)	−0.54	0.59
CFI	4.8 (2.6–6.9)	3.5 (2.5–4.8)	−1.45	0.15
TUG, seconds	6.5 (5.9–7.7)	5.7 (5.2–6.8)	−1.8	0.066
Grip strength, kg	23.7 (22.4–29.1)	27.6 (21.1–32.6)	−0.45	0.65
GDS	3.0 (2.3–5.5)	3.0 (1.5–5.5)	−0.31	0.76
EQ‐5D	0.895 (0.774–0.938)	0.895 (0.847–0.938)	−0.37	0.71
PASE	115.7 (83.3–175.6)	104.8 (64.7–158.8)	−0.65	0.52

Data are presented as median (25th—75th percentile). *P*‐values are calculated using the Mann–Whitney *U*‐test. BTG, booster training group; NonBTG, non‐booster training group; MMSE, Mini‐Mental State Examination; Five Cog, Five Cognitive Functions Test; CFI, Cognitive Function Instrument; TUG, Timed Up & Go Test; GDS, Geriatric Depression Scale; PASE, Physical Activity Scale for the Elderly.

### Effects of the intensive training and the booster training

Regarding the tests for cognitive function, the MMSE (*P* = 0.047) and the subtests of the Five Cog (attention (*P* < 0.0001), memory (*P* < 0.0001), language (*P* = 0.035) and reasoning (*P* = 0.025)) showed significant improvement after intensive training. The TUG test time for evaluating physical performance also improved significantly (*P* < 0.0001). We did not find any improvement in CFI, grip strength, GDS, EQ‐5D and PASE (Table 2).

**Table 2:** The effects of the intensive training

	Baseline	PIT	Z score	*P*‐value
MMSE	27 (25—28)	28(26—29)	−1.99	0.047
Five Cog				
Attention	22(19—25.5)	28(23—30)	−5.49	<0.0001
Memory	12(9—15.5)	15(11—20.5)	−5.31	<0.0001
Visuospatial	7(7—7)	7(7—7)	−0.425	0.67
Language	15(13—17)	16(13—19)	−2.11	0.035
Reasoning	11(8—12)	11(8—13)	−2.24	0.025
CFI	4.5(3—6.5)	4.0(2.5—6.5)	−1.63	0.10
TUG, seconds	7.2(6.4—8.1)	6.0(5.5—7.2)	−5.41	<0.0001
Grip strength, kg	23.6(20.1—31.8)	23.7(20.9—30.4)	−0.464	0.64
GDS	4(2—6)	3(2—6)	−0.132	0.90
EQ‐5D	0.895(0.8075—0.895)	0.895(0.815—0.9384)	−0.639	0.52
PASE	127.7(68.3—157.0)	113.9(79.8—168.0)	−0.84	0.40

Data are presented as median (25th—75th percentile). *P*‐values are calculated using the Mann–Whitney *U*‐test. PIT, post‐intensive training; MMSE, Mini‐Mental State Examination; Five Cog, Five Cognitive Functions Test; CFI, Cognitive Function Instrument; TUG, Timed Up & Go Test; GDS, Geriatric Depression Scale; PASE, Physical Activity Scale for the Elderly.

Our analysis of the effects of the booster training revealed no additional significant differences in most of the outcome measures. Although there was a significant interaction in the CFI (*P* = 0.006) and the TUG (*P* < 0.0001) (Table 3), the pairwise comparison did not reveal any significant differences.

**Table 3:** The effects of the booster training

							Mixed effect model		
	Baseline	PIT	FU‐1	FU‐2	FU‐3	FU‐4	Group	Time	Interaction
MMSE									
BTG	27.2 (1.9)	27.5 (1.9)	28.3 (1.3)	28.3 (1.5)	28.2 (1.5)	27.6 (1.6)	0.71 (0.17)	0.97 (0.0055)	0.92 (0.020)
NonBTG	27.4 (1.7)	28.2 (1.7)	28.2 (1.6)	28.4 (1.7)	27.8 (2.3)	28.5 (1.8)			
Five Cog									
Attention									
BTG	24.2 (5.9)	27.9 (5.2)	25.8 (7.1)	24.8 (7.2)	24.6 (5.9)	24.6 (7.9)	0.62 (−1.0)	0.001 (−0.73)	0.86 (−0.05)
NonBTG	20.4 (7.7)	27.1 (5.1)	23.8 (6.2)	25.2 (6.6)	23.8 (6.0)	23.3 (5.8)			
Memory									
BTG	13.3 (4.5)	15.9 (6.1)	16.1 (5.9)	17.2 (7.1)	17.1 (7.1)	17.7 (7.4)	0.83 (0.52)	0.010 (0.44)	0.20 (−3.0)
NonBTG	13.1 (5.2)	16.5 (6.8)	16.7 (7.1)	16.7 (6.9)	16.6 (7.4)	17.3 (6.9)			
Visuospatial									
BTG	6.9 (0.34)	6.9 (0.25)	6.9 (0.25)	6.9 (0.25)	6.9 (0.25)	6.9 (0.25)	0.084 (−0.18)	1.0 (<0.001)	0.087 (0.070)
NonBTG	6.7 (0.59)	6.6 (1.7)	6.8 (0.44)	6.9 (0.24)	7 (0)	7 (0)			
Language									
BTG	16.8 (2.4)	18.4 (4.2)	16.8 (3.2)	18.0 (3.7)	18.6 (4.1)	18.3 (3.5)	0.51 (−0.96)	0.45 (0.14)	0.93 (0.023)
NonBTG	16.5 (4.2)	16.6 (4.0)	16.8 (4.2)	17.7 (5.3)	18.1 (5.8)	17.2 (5.7)			
Reasoning									
BTG	10.2 (3.2)	11.6 (2.5)	10.9 (2.5)	11.1 (3.1)	10.9 (3.0)	10.9 (3.1)	0.81 (0.22)	0.24 (−0.13)	0.79 (−0.04)
NonBTG	11.4 (2.7)	11.3 (2.5)	11.8 (2.6)	11.5 (2.4)	10.9 (2.8)	10.8 (2.9)			
CFI									
BTG	5.1 (2.9)	5.2 (2.6)	4.2 (2.4)	4.3 (2.3)	4.3 (2.7)	4.2 (2.2)	0.22 (−0.89)	0.085 (−0.17)	0.006 (0.39)
NonBTG	4.9 (2.2)	3.8 (2.2)	4.1 (2.0)	4.4 (1.9)	4.4 (2.1)	4.7 (2.0)			
TUG, seconds									
BTG	7.8 (1.4)	7.0 (1.6)	7.4 (1.3)	7.0 (1.4)	6.8 (1.5)	6.5 (1.3)	0.034 (−0.98)	0.005 (−0.15)	0.000 (0.30)
NonBTG	7.1 (1.4)	6.0 (1.3)	6.9 (1.7)	6.6 (1.5)	6.8 (1.7)	6.8 (1.7)			
Grip strength, kg									
BTG	25.0 (6.3)	25.4 (5.3)	24.4 (6.1)	24.1 (5.9)	24.2 (5.5)	24.7 (5.5)	0.29 (2.3)	0.17 (−0.16)	0.15 (−0.24)
NonBTG	27.3 (7.7)	27.6 (7.6)	26.9 (7.5)	27.2 (7.3)	26.0 (7.5)	26.1 (7.6)			
GDS									
BTG	4.7 (3.7)	4.2 (3.0)	4.9 (2.6)	3.1 (2.6)	4.4 (3.3)	3.6 (3.6)	0.36 (−0.77)	0.032 (−0.25)	0.089 (0.27)
NonBTG	3.5 (2.2)	3.8 (3.0)	3.7 (2.3)	3.4 (2.0)	3.7 (2.5)	3.9 (2.6)			
EQ‐5D									
BTG	0.787 (0.16)	0.84 (0.12)	0.83 (0.11)	0.843 (0.10)	0.821 (0.13)	0.854 (0.13)	0.16 (0.042)	0.54 (0.0028)	0.068 (−0.012)
NonBTG	0.866 (0.089)	0.87 (0.081)	0.88 (0.070)	0.876 (0.059)	0.838 (0.091)	0.844 (0.086)			
PASE									
BTG	95.1 (42.1)	124.9 (56.0)	98.3 (46.4)	102.7 (43.3)	97.3 (63.3)	110.4 (72.6)	0.47 (13.3)	0.16 (−3.9)	0.64 (1.8)
NonBTG	142.9 (72.1)	121.2 (70.9)	131.9 (65.2)	127.7 (62.4)	122.3 (51.6)	112.7 (47.3)			

Data (baseline, PIT, FU‐1,2,3,4) are presented as mean (SD). The results of the mixed effect model are shown as *P*‐values (estimate values). BTG, booster training group; NonBTG, non‐booster training group; PIT, post‐intensive training; FU‐1, follow up 1; FU‐2, follow up 2; FU‐3, follow up 3; FU‐4, follow up 4; MMSE, Mini‐Mental State Examination; Five Cog, Five Cognitive Functions Test; CFI, Cognitive Function Instrument; TUG, Timed Up & Go Test; GDS, Geriatric Depression Scale; PASE, Physical Activity Scale for the Elderly.

## DISCUSSION

### Short‐term effects of the intensive training

In this study, we observed positive effects on cognitive function in the MMSE and the Five Cog assessments (excluding ‘visuospatial’), although the CFI did not show any significant changes. MMSE showed a statistically significant improvement, but the score changed only by 1 point, showing no great clinical impact. There were 10 sessions of intensive training, carried out weekly, and the period between baseline and PIT assessment was only approximately 3 months. The result of the intensive training was the short‐term effect of the DPP.

The training sessions mainly combined physical exercise and cognitive training; many other studies have shown the effects of each of these. A systematic review^21^ suggested that physical exercise can improve cognitive function. Erickson^22^ reported that physical exercise, particularly aerobic exercise, helped increased blood flow and metabolism in the frontal lobe and the hippocampus; in addition, increased hippocampal volume was associated with greater serum levels of brain‐derived neurotrophic factor, a mediator of neurogenesis in the dentate gyrus. Lazarov^23^ also found that exposure of transgenic mice to a stimulating environment resulted in pronounced reductions in cerebral ABeta levels and amyloid deposits, compared to animals raised under standard conditions. In the present study, the TUG score improved significantly after intensive training, which shows that the physical exercise involved in the intensive training enhanced the level of fitness of the participants. As there was no change in the PASE, we considered that it was not possible to affect the daily living activities of the participants, but weekly training did affect their physical and cognitive functioning.

Conversely, the results of cognitive training studies have indicated that training for executive functions (e.g., working memory) increases the efficiency of the prefrontal network, which provides support for brain functioning in the face of cognitive decline. Physical activity preserves neuronal structural integrity and brain volume, whereas cognitive activity strengthens the functioning and plasticity of neural circuits.^24^, ^25^ However, some studies have reported cognitive training to be ineffective. Owen^26^ performed cognitive training with more than 10 000 subjects and found that the cognitive improvement in the intervention group was not significant compared to that in the control group. In the present study, the DPP was implemented through a combination of physical exercise and cognitive training, and it is therefore not clear which program had a greater effect on the participants' cognitive functioning. Effective programs may vary depending on the physical and cognitive functions of the participants at baseline. Therefore, when implementing a DPP for many people simultaneously, it is better to perform multimodal training that includes elements of both exercise and cognition.^27^

### The effects of the booster training

Intensive training increased the scores for cognitive and physical functioning, but booster training had no significant effects on the psychosomatic functioning of the subjects in terms of changes both within and between the groups. The purpose of the 3‐monthly booster training was not so much as the direct effect of the training program itself but more to raise the participants' awareness of dementia prevention by training regularly. Raising awareness may lead to behaviour modification that promotes the health of the elderly in daily life. Exercises in daily life, particularly walking outside, can be a chance to communicate with one's neighbours. It has been reported that cognitive function tends to be reduced in people with few social networks, and a decrease in participation in social activity increases the risk of developing dementia.^28^, ^29^ However, in the present study significant changes from either intensive training or booster training could not be confirmed in the PASE, which might be related to there being no change in either the GDS or the EQ5D. According to Fleg,^30^ regular exercise has been shown to reduce symptoms of depression and enhance total quality of life; thus, there is a close relationship between the amount of activity performed in daily life and the psychological aspects of the elderly. For people to voluntarily continue activities to prevent dementia, it is necessary to raise awareness of behaviour change in respect of the psychological aspects of the elderly. A previous interventional study^31^ found that a 6‐month program of walking twice a week slowed cognitive decline compared to a control group. Although we conducted booster training every 3 months in the BTG, this frequency may have been too low to achieve further improvement in cognitive‐healthy elderly individuals, excluding those with MCI.

During the follow‐up period after the intensive training, neither group displayed a significant improvement at each evaluation. In a prospective observational study for the cognitively intact elderly (≦ 70), the MMSE scores of 16.9% of the participants fell by three points or more during the follow‐up period (average period of 2.7 years).^32^ However, in the present study we found that the MMSE score of only one participant (3.3%) fell by three points or more during the survey period of approximately two and a half years. The developmental trajectory of cognitive ability, involving novel problem solving, spatial manipulation, mental speed and identifying complex relations among stimulus patterns, is thought to follow neurological maturation, peaking in the mid‐20s and declining gradually until the 60s, after which there is a more rapid decline.^33^ Given that the mean age of our subjects was 76.4 years at baseline assessment, being able to maintain test scores for cognitive ability can be seen as a positive effect of the DPP, especially the intensive training. Actually, there is a large cost involved for a municipality in continuous implementation of a DPP for a large number of residents. In the present study, although we found no effects of the 3‐monthly booster training, the functions of the elderly participants were maintained for approximately two and a half years. In other words, we can state that conducting intensive training without follow‐up training might be of some positive effect in preventing dementia in community‐dwelling elderly.

This study was an exploratory study with a limited sample size, and the participants included cognitive‐healthy elderly individuals, excluding those with MCI. Therefore, the DPP effect on the incidence of dementia has not been clarified in this study. Although the booster training was performed on a 3‐monthly basis, the question of whether the municipality can bear the cost of such training should be considered, and it is possible that the effect may differ if the frequency is changed. Further research is required to develop a realistic and effective DPP.

## CONCLUSION

The intensive DPP might be useful for improving or maintaining the cognitive and physical functions of cognitive‐healthy elderly individuals. Although the effect of the booster training could not be verified, the physical and cognitive functions of the elderly participants were maintained for approximately two and a half years. These findings support conducting intensive training without follow‐up training, as this might have some effect in preventing dementia in the community‐dwelling elderly.
